# Deconstructing Alzheimer’s Disease: How to Bridge the Gap between Experimental Models and the Human Pathology?

**DOI:** 10.3390/ijms22168769

**Published:** 2021-08-16

**Authors:** Anaïs Vignon, Lucie Salvador-Prince, Sylvain Lehmann, Véronique Perrier, Joan Torrent

**Affiliations:** 1 INM, University of Montpellier, INSERM, 34095 Montpellier, France; anais.vignon@umontpellier.fr (A.V.); lucie.salvador-prince@umontpellier.fr (L.S.-P.); 2 INM, University of Montpellier, INSERM, CHU Montpellier, 34095 Montpellier, France; sylvain.lehmann@umontpellier.fr; 3 INM, University of Montpellier, INSERM, CNRS, 34095 Montpellier, France

**Keywords:** Alzheimer’s disease, in vitro, in cellulo, in vivo models

## Abstract

Discovered more than a century ago, Alzheimer’s disease (AD) is not only still present in our societies but has also become the most common dementia, with 50 million people worldwide affected by the disease. This number is expected to double in the next generation, and no cure is currently available to slow down or stop the disease progression. Recently, some advances were made due to the approval of the aducanumab treatment by the American Food and Drug Administration. The etiology of this human-specific disease remains poorly understood, and the mechanisms of its development have not been completely clarified. Several hypotheses concerning the molecular mechanisms of AD have been proposed, but the existing studies focus primarily on the two main markers of the disease: the amyloid β peptides, whose aggregation in the brain generates amyloid plaques, and the abnormally phosphorylated tau proteins, which are responsible for neurofibrillary tangles. These protein aggregates induce neuroinflammation and neurodegeneration, which, in turn, lead to cognitive and behavioral deficits. The challenge is, therefore, to create models that best reproduce this pathology. This review aims at gathering the different existing AD models developed in vitro, in cellulo, and in vivo. Many models have already been set up, but it is necessary to identify the most relevant ones for our investigations. The purpose of the review is to help researchers to identify the most pertinent disease models, from the most often used to the most recently generated and from simple to complex, explaining their specificities and giving concrete examples.

## 1. Introduction

Alzheimer’s disease (AD) is the most common form of dementia in humans. It is a health and social issue that causes great economic concerns, with 131.5 million people around the world predicted to be affected in 2050 [1]. AD is a multifactorial disease with genetic and environmental risk factors. Two forms of the disease exist: (i) the familial AD (fAD), affecting less than 1% of the patients [2] and caused by one or several mutations among the three main AD-related genes, and (ii) the sporadic AD (sAD), which is the most prevalent form associated with genetic predispositions and environmental factors, such as age, gender, alimentation, and pollutant exposure [3]. When symptoms occur, the age of the patient is a key factor in defining the type of AD. Between 40 and 65 years of age, the disease is considered as an early onset of AD (EOAD), and after 65 years of age, it is considered as a late onset of AD (LOAD). The EOAD accounts for less than 5% of all AD cases [4] and is preferably linked to fAD, with an early development induced by a genetic mutation [5]. The main hallmarks of AD in the brain are an extracellular accumulation of amyloid plaques due to an aggregation of amyloid β (Aβ) peptides and an intracellular accumulation of neurofibrillary tangles (NFTs) formed by hyperphosphorylated tau proteins. These protein assemblies induce neuroinflammation and neurodegeneration [6]. The affected brain regions are the cortex, the temporal, parietal, and frontal lobes, and the hippocampus [7]. Neurodegeneration in these regions, especially of cholinergic neurons, leads to memory and cognitive impairments [5]. AD is a complex disease due to the interaction of many actors, combined with the difficulty to identify those responsible for the onset of the disease. Furthermore, AD is associated with other severe human diseases, such as hypertension, glucose metabolism abnormalities, and diabetes [8].

Since the description of the first case of AD by Alois Alzheimer more than a century ago [9], the pathogenesis of the disease has still not been clearly understood. In the mid-1970s, the cholinergic hypothesis was proposed on the basis that patients exhibited low levels of acetylcholine. The activity of choline acetyltransferase, a key enzyme in acetylcholine synthesis, was greatly reduced in different brain regions of AD patients, i.e., the amygdala, hippocampus, and cortex. The concept of a cholinergic system failure in AD was reported [10]. This hypothesis was the starting point for the development of many inhibitors of acetylcholinesterase (AChE), the enzyme involved in acetylcholine degradation. Among those inhibitors, tacrine was the first drug approved by the American Food and Drug Administration (FDA) in 1995 [11]. However, its prescription has dropped due to liver toxicity and the appearance of a second generation of AChE inhibitors: donepezil, rivastigmine, and galantamine [12]. In parallel, the *N*-methyl-d-aspartate (NMDA) hypothesis was proposed in 1992 on the basis that Aβ peptides alter calcium homeostasis by interacting with NMDA receptors and render neurons sensitive to excitotoxicity. Thus, antagonists of NMDA receptors were developed, such as memantine, which was approved by the American FDA in 2003 [13]. However, both types of drugs only lessen the symptoms, such as memory loss, for a very limited period [14]. Besides, in 2012, the French Pharmacoeconomic Committee downgraded both treatments from major to low efficiency. After many infructuous trials, there is still no efficient treatment to cure or stop the progression of cognitive impairments. In this context, the development of AD experimental models is crucial for elucidating the mechanisms and the etiology of the disease in order to prevent its development, allow for early diagnoses, and possibly identify efficient therapies [3].

Here, we aim at presenting an overview of the main existing AD experimental models and their features. After the identification of amyloid plaques and neurofibrillary tangles as the main targets of AD pathology, the models focused on the formation of Aβ and tau aggregates in vitro. Recent breakthroughs in structural techniques have allowed for the characterization, at the atomistic level, of Aβ and tau assemblies. This, together with the improvement of in silico models, has helped to identify new pharmacological targets. In parallel, in cellulo and in vivo models overexpressing Aβ or tau markers were extensively generated. However, the discrepancies between in vivo models and the human pathology, combined with the successive failures in clinical trials, led to the development of new models mimicking the brain environment: induced pluripotent stem cells (iPSCs) derived from AD patients, 3D-cultures, and organoids/mini brains. This review does not provide an exhaustive list of all the models, because they are too numerous; rather, it selects the most representative and recent models using the latest innovative technologies.

## 2. Molecular Features of Alzheimer’s Disease: Aβ Peptides and Tau Proteins

**Figure 1:**
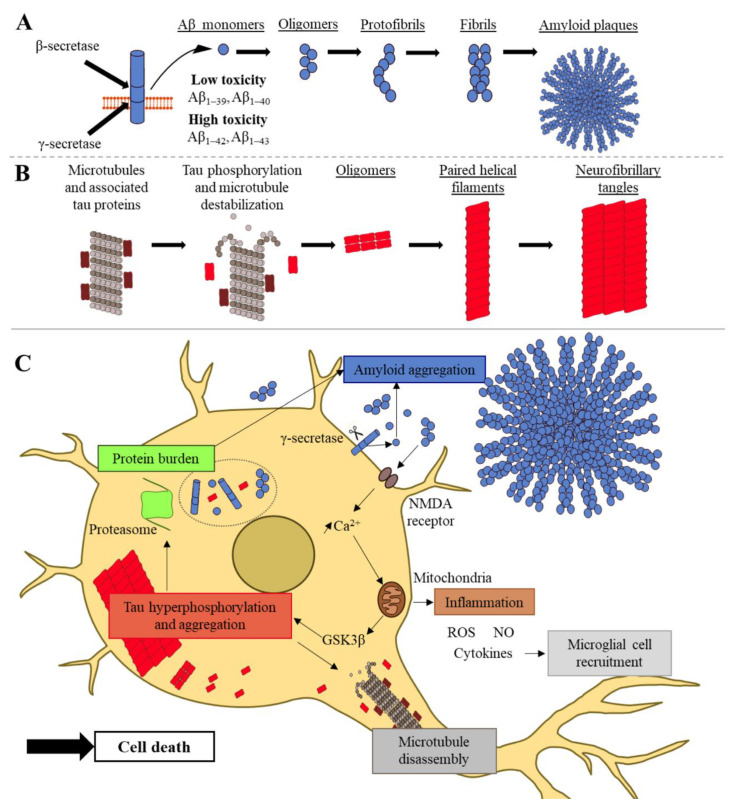
Molecular features of Alzheimer’s disease: from amyloid and tau aggregation to cell death. (**A**) Amyloid peptide production and aggregation. The APP transmembrane protein (blue cylinders) is cleaved by β- and γ-secretases, which generate Aβ peptides (blue circles). In a pathological context, these Aβ peptides aggregate into toxic oligomers, which form protofibrils, fibrils, and finally, amyloid plaques. (**B**) Tau aggregation. Tau proteins (dark red rectangles) are associated with microtubules (gray assemblies). During the pathological process, tau proteins are hyperphosphorylated (light red rectangles) and detach from microtubules, which disassemble during the pathological process. Hyperphosphorylated tau proteins aggregate into oligomers, forming paired helical filaments and neurofibrillary tangles (NFTs). (**C**) Summary of the molecular interactions occurring in a neuron during AD and illustration of a tau–Aβ vicious circle. On one hand, Aβ oligomers increase intraneuronal calcium, inducing inflammation, microglial recruitment, and tau aggregation. On the other hand, the hyperphosphorylation of tau induces microtubule disassembly and tau aggregation. In turn, the excess of tau aggregates decreases Aβ peptide degradation by disturbing the proteasome. All these processes lead to neuronal cell death. Abbreviations. NMDA: *N*-methyl-d-aspartate; GSK3β: glycogen synthase kinase 3 beta; ROS: reactive oxygen species; NO: nitric oxide.

Today, the amyloid and tau pathways, based on the involvement of the Aβ peptides and tau protein, respectively, are the two hypotheses related to the development of the AD pathology. Amyloid plaques are composed of amyloid fibrils formed by the aggregation of Aβ peptides (Figure 1A). These peptides are produced by several key actors: neurons and astrocytes, which are inter-connected [15,16]. From 36 to 43 amino acids in length, Aβ peptides are cleavage products of the transmembrane amyloid precursor protein (APP). APP can be cleaved by three types of enzymes: α-, β-, and γ-secretases. The α-secretases are enzymes from the ADAM (A Desintegrin and Metalloprotease) family, such as ADAM9, ADAM10, and ADAM17 [17]. The β-secretase is the β-site APP cleaving enzyme (BACE1) [18]. The γ-secretase is a protein complex composed of presenilin 1 (PSEN1) or presenilin 2 (PSEN2), nicastrin, anterior pharynx defective 1 (APH1), and presenilin enhancer 2 (PEN2). In the physiologic nonamyloidogenic pathway, APP is mostly cleaved by the α- and γ-secretases. In the amyloidogenic pathway, the cleavages of APP by β- and γ- secretases lead to the extracellular production of Aβ peptides, especially the insoluble 42-amino-acid peptide, Aβ_1–42_ [5]. The latter is the main component of amyloid plaques and is known as the most toxic AD molecule when aggregating and forming oligomers. As a protection mechanism, amyloid fibrils could trap toxic oligomers. The toxicity mechanisms of oligomers are based on their interaction with different cellular receptors, such as nerve growth factor (NGF), NMDA, insulin, and Frizzled receptors [19]. For instance, when Aβ oligomers interact with NMDA receptors, the calcium homeostasis is impaired. This calcium imbalance alters mitochondrial functions, inducing the production of reactive oxygen species (ROS) and nitric oxide (NO) (Figure 1C). These processes lead to neuroinflammation and cytokine release, with the recruitment of microglial cells and astrocytes, as well as a decrease of neurotransmission. The inflammatory environment and the neurotransmission dysregulation cause neurotoxicity and neurodegeneration [6]. In the amyloid hypothesis, an excess of Aβ_1–42_ peptides is the cause of AD development. Knowing that mutations in the *APP*, *PSEN1*, or *PSEN2* genes lead to fAD, this means that APP and β- and γ-secretases are key actors in AD development and supports the hypothesis that the dysregulation of Aβ peptide production could be a cause of AD. However, fAD cases represent a minority among all affected patients. A comparison of the Aβ production of healthy controls and that of AD patients suggests that it is not only a production problem but also a decrease or a dysregulation of the Aβ peptide clearance [20]. Aβ peptides are mainly degraded by enzymes, such as neprilysin (NEP) and insulin-degrading enzyme (IDE), or eliminated from the brain by crossing the blood–brain barrier (BBB) through the lipoprotein receptor-related protein-1 (LRP-1) receptor. On the other hand, Aβ peptides can be transported from blood to the central nervous system via the receptor for advanced glycation end products (RAGE) [21]. The BBB is composed of endothelial cells, pericytes, and astrocytes, which form the neurovascular unit interacting with neurons and microglia [5,22]. These different actors have a role in Aβ peptide regulation and AD development (reviewed by Bates et al. [21]).

NFTs are composed of the hyperphosphorylated microtubule-associated protein tau (MAPT), which is also called tau (Figure 1B). This protein is usually associated with microtubules, which have an important role in the cell cytoskeleton. In AD, the balance between phosphatases and kinases is dysregulated, leading to tau hyperphosphorylation at certain sites. Hyperphosphorylated tau proteins detach from the microtubules and accumulate into the cytosol, forming paired helical filaments and straight filaments, which assemble into NFTs [23,24]. NFTs lead to microtubule dysfunction, with an axonal transport alteration and cytoskeleton breakdown, inducing neuronal death. Tau aggregation is also known to decrease protein degradation by altering proteasome and to induce immune response by activating microglia and astrocytes [5,25,26]. Thus, tau creates a protein burden, possibly inducing a higher Aβ quantity, and induces neurodegeneration and neuroinflammation (Figure 1C). In the tau hypothesis, the etiology of AD is related to the hyperphosphorylation of tau and its aggregation. However, many studies have shown that Aβ production is upstream of tau. In the amyloid hypothesis, the increase in intraneuronal calcium activates glycogen synthase kinase 3 beta (GSK3β), a kinase involved in tau hyperphosphorylation [27]. Thus, the dysregulation of Aβ peptide production or clearance also induces cascade events, leading to tau hyperphosphorylation and sustaining a tau–Aβ vicious circle (Figure 1C). Currently, the main hypothesis of AD development is the amyloid hypothesis, although it has been criticized due to a lack of correlation between Aβ plaques and neuronal loss [28]. However, the neuronal loss is caused by the presence of toxic Aβ oligomers, rather than fibrils and plaques, and it was suggested that AD is an “oligomeropathy” with a prion-like mechanism of propagation [3,29]. The hypothesis that the molecular interplay between tau and Aβ oligomers perpetuates a vicious circle of neurotoxicity is now more widely accepted, even though it remains to be elucidated whether tau or Aβ species initiate the pathological process [30,31].

## 3. Cell-Free In Vitro and In Silico Models of Alzheimer’s Disease

In this section, cell-free in vitro and in silico models using Aβ peptides and tau proteins are presented. These models investigate aggregation kinetics, protein structures, and molecular interactions with potential ligands. They are diverse and rely on many techniques. Since in vitro models are at the molecular scale, with a limited number of molecular players, they are not able to precisely predict the effects on the entire organism. However, they are at the starting point to explore and develop biological, pharmacological, and medical research on AD.

### 3.1. In Vitro Aβ Models of Fibrillization—Monitoring the Kinetics of Fibril Formation Using Biochemical and Biophysical Methods

#### 3.1.1. Dye-Based Methods

**Figure 2:**
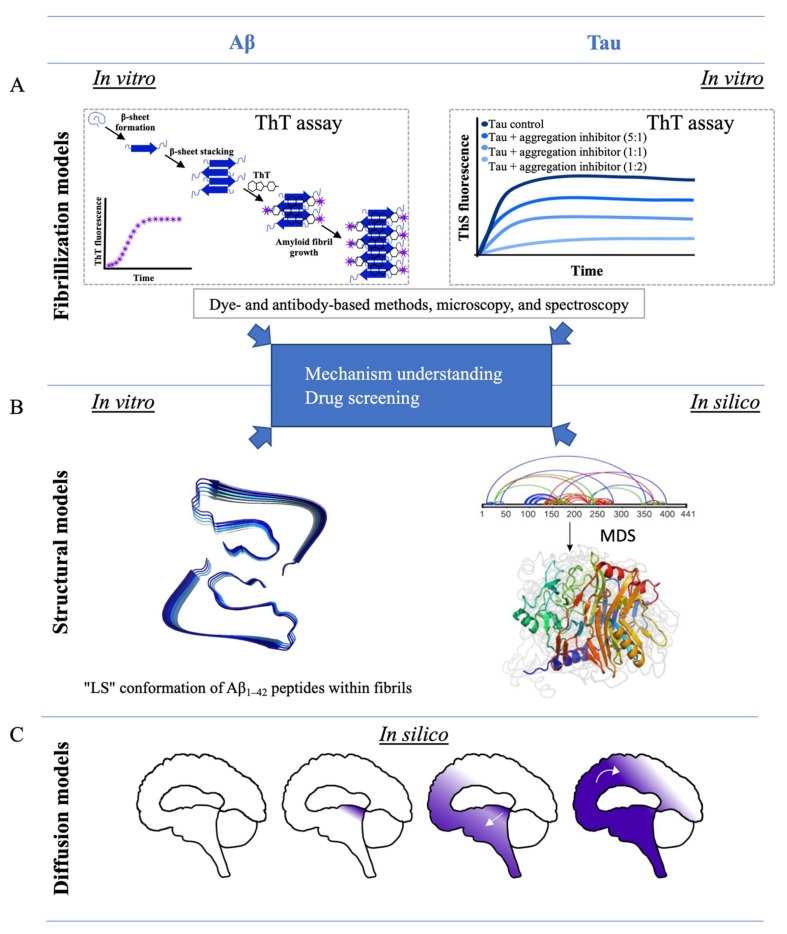
Cell-free in vitro and in silico models of Aβ peptides and tau proteins. (**A**) Fibrillization models of Aβ peptides and tau proteins based on ThT assays. (**B**) Structural models of Aβ_1–42_ peptides and 441-residue tau protein by Gremer et al. (2017) [57] and Popov et al. (2019) [58], respectively. (**C**) Models of protein diffusion of Aβ peptides and tau proteins in the brain developed by Weickenmeier et al. (2018) [59] and Yang et al. (2019) [60]. Abbreviations. ThT: Thioflavin T; MDS: molecular dynamic simulations.

Since the 1990s, the development of in vitro models of Aβ peptide aggregation has been a limited but crucial step in understanding the molecular mechanisms underlying AD. Aβ peptides are self-aggregating molecules in vitro. Their aggregation is due to the formation of β-sheets in their secondary structure. To monitor the fibrillization process, the first in vitro experiments used dyes, which are colored compounds binding specific substrates. Congo Red (CR) was the first compound to be used [32] and was shown to bind to β-pleated sheets, identifying fibrils but not protofibrils and oligomers (Table 1). In 1959, another fluorescent dye was developed: thioflavin T (ThT) [33]. Through the binding of ThT to β-pleated structures of amyloid fibrils, its fluorescence emission intensity increases. Even if ThT does not bind to protofibrils and oligomers, it is still the best and most commonly used fluorescent dye for studying the kinetics of fibril formation (Figure 2) [34]. The binding mechanism of ThT to fibrils is not clearly understood. In addition, a major disadvantage is that ThT also binds to DNA. Thus, it is crucial to use protein-pure samples in in vitro models of fibrillization. Another more recent fluorescent dye for studying amyloid aggregation is 1-anilino-8-naphthalene sulfonate (ANS) [35,36], which binds to solvent-exposed hydrophobic areas of proteins and induces a fluorescence emission shift. Thus, ANS is very useful for protein folding studies and for staining amyloid intermediates, such as oligomers and protofibrils. In contrast to CR and ThT, ANS allows for the characterization of both the early (oligomers, protofibrils) and late steps of aggregation (fibrils).

#### 3.1.2. Antibody-Based Methods

Alternatively, specific antibodies/nanobodies are now powerful tools for precisely targeting the various species involved in the aggregation process of Aβ peptides. The homogeneous time-resolved fluorescence (HTRF) immunoassay is based on the fluorescence resonance energy transfer (FRET) between two antibodies, i.e., the donor and the acceptor, which are directed against different parts of the fibrils. During the fibrillization process, when the donor antibody is close enough to the acceptor, it transfers its energy, leading to the emission of a fluorescence signal. Thus, the HTRF immunoassay resolves aggregation kinetics in real-time [44,45] (Table 1). Surface plasmon resonance (SPR) is another antibody-based method that allows for interaction studies of partners. It was adapted for analyses of Aβ aggregation kinetics. This latter method is a label-free and real-time technique measuring the short-term (seconds) and long-term (hours) kinetics of amyloid aggregation [46,47].

#### 3.1.3. Microscopy and Spectroscopy Techniques

With the development of high-performance microscopes and specific fluorophores, new in vitro models of fibrillization were developed based on fluorescence correlation microscopy (FCS). FCS uses fluorophores that specifically bind to amyloid peptides [50,51] (Table 1). For example, Sengupta et al. [42] studied the saturation concentration under which Aβ_1–40_ peptides do not precipitate in vitro. This measured concentration, at a micromolar range, is higher than the estimated in vivo saturation concentration at a nanomolar range. The discrepancies between the two concentrations highlight the role of co-precipitation factors promoting amyloid aggregation at nanomolar concentrations in vivo, rather than the self-aggregating properties of amyloid peptides alone.

One of the main pitfalls of the in vitro models described above is the use of extraneous molecules, i.e., dyes, antibodies, or fluorophores, in the experiments. These molecules are necessary for studying fibril formation kinetics; however, they are not present in vivo and could interfere with the fibrillization modeling. Therefore, highly pure in vitro models, without the addition of external molecules, were developed. For example, a recent in vitro model, based on the time-resolved emission spectra (TRES), uses the intrinsic tyrosine fluorescence of amyloid peptides to monitor aggregation at a nanosecond timescale [53]. Tridimensional models of fibrils were also generated using light scattering and derived techniques, such as multiangle laser light scattering (MALLS) and dynamic light scattering (DLS). These techniques quantify the size of aggregates and allow for the monitoring of fibril formation through the detection, in real-time, of the different growing amyloid structures [37,38,55,56] (Table 1).

To summarize this section, in vitro amyloid models of fibrillization are based on very diverse techniques. This diversity has helped to elucidate the required parameters necessary to induce aggregation. Depending on the model, researchers focus either on the timescale, identifying the short-term [61] or long-term fibrillization events, or are interested in one specific amyloid species. For instance, Nick et al. [62] modeled oligomerization through a complete study of a fibrillization-resistant oligomer. These in vitro models of fibrillization are also very useful for finding inhibitors blocking the fibrillization or to discover ligands able to promote the kinetics. Above all, the closer the in vitro model is to reality, the more accurately researchers can understand the disease at the molecular level.

### 3.2. Structural Models of Aβ Amyloid Peptides

To deeply understand why amyloid peptides oligomerize and form fibrils and plaques, it is essential to determine their structures and interaction forces that govern the different structural species, i.e., monomers, oligomers, protofibrils, and fibrils. The determination of the structures is also a crucial step in performing high-throughput screening for the identification of specific ligands binding to amyloid structures and inhibiting the fibrillization process. Many attempts to generate Aβ amyloid structure models have been made, without success [63]. Only recently, the structure of an Aβ_1–42_-composed fibril was resolved at a resolution of 4 angstrom using cryo-electron microscopy (EM) and solid-state nuclear magnetic resonance (ssNMR) techniques. This structure gives the position of peptides inside the protofibrils and the position of intertwined protofibrils inside the fibrils (Figure 2). In this model, each Aβ_1–42_ peptide adopts a “LS” conformation and is not planar. Each peptide, in its “LS” conformation, interacts with nearby Aβ_1–42_ peptides from the same protofibril and also from the other intertwined protofibrils [57]. The structure of Aβ amyloid fibrils isolated from meningeal tissues of Alzheimer’s patients was also resolved recently by cryo-EM. The structural analysis showed differences between synthetic fibrils and patient-derived fibrils. These discrepancies underscore the relevance of using fibrils derived from patients, rather than recombinant fibrils generated from *Escherichia coli* [64]. Additionally, ssNMR, performed on tissue samples extracted from AD patients, revealed that structural variations in amyloid fibrils can be correlated with different clinical phenotypes, leading to the notion of AD strains. Some strains lead to a rapid AD pathology, whereas other strains induce a prolonged duration of AD [65].

Thus, in vitro Aβ structural models and Aβ fibrillization models are complementary. Most cell-free in vitro studies use both to understand the AD pathology. For example, a difference in the primary structure of one Aβ peptide, such as mutation, can lead to an increased aggregation rate, as demonstrated by He et al. [66], who compared two amyloid peptides carrying fAD mutations (the Dutch-type and its L17A/F19A-substituted Aβ_1–40_ mutant) with the native Aβ_1–40_ peptide.

### 3.3. Pharmacological Development Targeting Aβ Peptides

The in vitro fibrillization and structure models of Aβ are not only used to understand the AD pathology but are also useful for identifying new drugs for potential treatments of AD. One of the first strategies developed to identify anti-AD drugs was to block the fibrillization process, and screening was performed using in vitro fibrillization models. For example, the inhibition of Aβ aggregation by carbenoxolone was shown using the Congo Red assay [67]. However, among the different in vitro fibrillization models, the ThT-based assay is the most commonly used for drug screening. For example, many designed drugs (pegylated copolymer and synthesized liposomes), as well as natural compounds (plant extracts, such as polyphenols, and marine-derived carotenoids, such as astaxanthin and fucoxanthin) were identified using ThT assays [40,41,42,43,68]. The role of cellular actors, such as chaperone, in fibrillization inhibition was also examined using a ThT assay [69]. Another emerging idea about how to prevent AD is rather to avoid the formation of toxic oligomers. Therefore, instead of looking for fibrillization inhibitors, synthetic peptides were designed to trap Aβ_1–42_ in chimeric amyloid-like fibrils [70]. In addition, Nardo et al. [71] studied the ability of synthetic liposomes to hinder early Aβ oligomerization in vitro.

### 3.4. In Silico Studies of Aβ Peptides

In silico models of AD use computer simulations to create models that mimic in vivo or in vitro systems of the disease in a simplified environment with the minimum number of parameters. In silico models are often based on in vitro structural models, and the two fields are entangled. In most cases, in silico models focus on molecular interactions during amyloid aggregation. They are notably based on molecular dynamic simulations (MDS), which quantify all the energies of intra-atomic interactions of one folded molecule or several molecules interacting together to simulate the most probable structural evolution with time. The study of molecular interactions between a protein and a ligand, such as the amyloid peptide and an aggregation inhibitor, was also made possible by molecular docking [72,73]. This technique simulates the probable structure of the protein–ligand complex using the known structure of the protein and the ligand, alone. For all the different methods, before simulation, different parameters can be chosen and changed, such as the peptide concentration, temperature, and presence of ions. Thus, in silico simulations, such as in vitro models of fibrillization and molecular structures, are used to study amyloid aggregation and allow for the structure modeling of amyloid peptides, oligomers, and fibrils. The simulations are also used to screen interacting drugs or aggregative factors. The computer modeling is advantageous, as it is inexpensive, reproducible, and high-throughput [3]. In silico models are often complementary to in vitro studies. For example, structures of Aβ_1–40_ and Aβ_1–42_ peptide assemblies were studied using a combination of MDS and atomic force microscopy [74]. The specific S-shape conformation of Aβ_1–42_ fibrils was extensively studied in silico and in vitro, as reviewed by Villalobos Acosta et al. [75]. In silico models are also useful for selecting inhibitors of the aggregation reaction with the highest scores. This method is very helpful and timesaving in in vitro drug screenings [73]. For example, Nie et al. [76] studied the interaction of a polyphenol, gallic acid, with Aβ_1–40_ monomers in silico. Polyphenols are known to inhibit Aβ aggregation, with an anti-amyloidogenic effect, but the mechanism is unknown. The binding site between gallic acid and the Aβ_1–40_ peptide, as well as the interaction forces, were described, and it was shown that, through the interaction, gallic acid prevents the beta-sheet structure formation in Aβ_1–40_. Recently, a physics-based model was developed to explain the prion-like features of propagation and neurodegeneration by simulating protein aggregates spreading within brain structures [59]. Their models were notably based on the full brain geometry and axonal directions. Playing with different parameters, such as the initial seeding point, they were able to recapitulate the different stages of AD. Their spatial simulations of toxic protein propagation and atrophy correspond to patients’ data (Figure 2).

To summarize, in vitro fibrillization and structural models, as well as in silico models, play a decisive role in the understanding of amyloid aggregation in AD, and they are key steps in the development of therapeutic strategies. These models are not exclusive and can be used in combination to study all the features of a potent drug, each of those models presenting pros and cons. For example, Som Chaudhury et al. [43] studied a tripeptide-based polymeric inhibitor of amyloid aggregation with a ThT assay (fibrillization), infrared spectroscopy (structure determination), and in silico simulations.

### 3.5. In Vitro and In Silico Models of the Structure and Aggregation of Tau

Tau aggregates into NFTs and is the other key actor in AD. Tau and Aβ peptides present similarities during the aggregation process, such as the formation of β-sheet structures [24,77]. Thus, tau structural and aggregation models were also developed, based on some of the previously described techniques used for in vitro and in silico amyloid models.

#### 3.5.1. Fibrillization of Tau Proteins and Pharmacological Studies

Since the ThT assay is based on the detection of beta-pleated structures, this test is also used for the study of tau aggregation and ultimately for drug screening [78]. In contrast to Aβ peptides, tau is not self-aggregating in vitro. Some intrinsic factors are required to induce its fibrillization, such as hyperphosphorylation, posttranslational modifications, preformed seeds, or the use of extrinsic inducers as heparin [79,80]. Curiously, lots of in vitro tau studies focused on proaggregation molecules to improve the aggregative models. Therefore, arachidonic acid was shown to induce and improve tau aggregation, especially for small tau isoforms [81]. The addition of an inducer into the test tube generates a bias in the model, since the tau aggregation rate is different with self-assembled phosphorylated tau, compared to heparin-induced tau [79]. Besides, there are different tau isoforms, which are all involved in the formation of NFTs in AD [82]. Thus, it is key to understand the aggregation process of each isoform and their interconnections to better understand their association within NFTs. This knowledge is a limiting step for the development of more specific drug screening assays [79]. In a similar way to the ThT assay, 200,000 compounds were screened, leading to the identification of anthraquinones as inhibitors of tau aggregation [83]. In addition, cinnamon, curcumin, and synthetic peptides also inhibited tau aggregation in ThT assays [84,85,86]. Recently, the microtubule polymerization assay, based on the turbidity of a tau-induced microtubule assembly, was developed. If the turbidity increases, this means that the microtubule assembly is fully functional. This assay is useful for assessing the physiological function of tau in microtubule assembly with the presence of drugs inhibiting tau aggregation [87].

#### 3.5.2. Structural Models of Tau Proteins

In vitro structural models of tau proteins are required to understand their molecular interactions during aggregation. Dregni et al. [88] used ssNMR to study the structure of heparin-induced tau fibrils composed of the full-length 4R tau isoform. 4R means that the isoform has four repeats of microtubule-binding domains. In this study, ssNMR showed that the protein core is rigid and that some parts of the 3D protein structure are dynamic. Comparing the tau structures from different patients, with either sporadic or familial AD, is a key step in understanding if differences at the molecular and structural levels correlate with physiopathological phenotypes [23].

#### 3.5.3. In Silico Studies of Tau Proteins

In silico modeling also helps in the establishment of structural models. Using discrete MDS, Popov et al. [58] realized the de novo structural determination of the 441-residue tau protein, detecting the β-sheet structures in the aggregation-prone regions (Figure 2). In silico structural models are used to simulate molecular interactions, especially with potential aggregation inhibitors. In a recent study, molecular simulations, molecular docking, and fragment molecular orbital calculations were performed to observe the interaction of tau with curcumin derivatives [89]. All these aggregation models and structures can be combined to elucidate the interactions of tau fibrils with their inhibitors. For instance, purpurin was identified as a potential tau inhibitor by a ThT-like assay and further characterized by circular dichroism and transmission EM, as well as molecular docking and molecular dynamic simulation [90].

Another interesting new model for understanding AD development was recently made by Yang et al. [60]. This in silico model of protein network diffusion in the brain was based on data of brain regions where tau is aggregated, as well as on data of the brain’s structural connectivity, recorded with diffusion tension imaging. This model simulated the diffusion of tau aggregates into a modeled brain by comparison to the spreading of tau in the patient’s brain. This innovative model can predict the evolution of the disease, allowing for the understanding of tau aggregation parameters and tau diffusion in the brain. Even if this model has pitfalls, such as a decrease of accuracy with the simulation time, this innovative in silico model is a pioneer in understanding the propagation of the protein aggregates leading to neurodegeneration (Figure 2).

## 4. In Cellulo Models of AD

**Figure 3:**
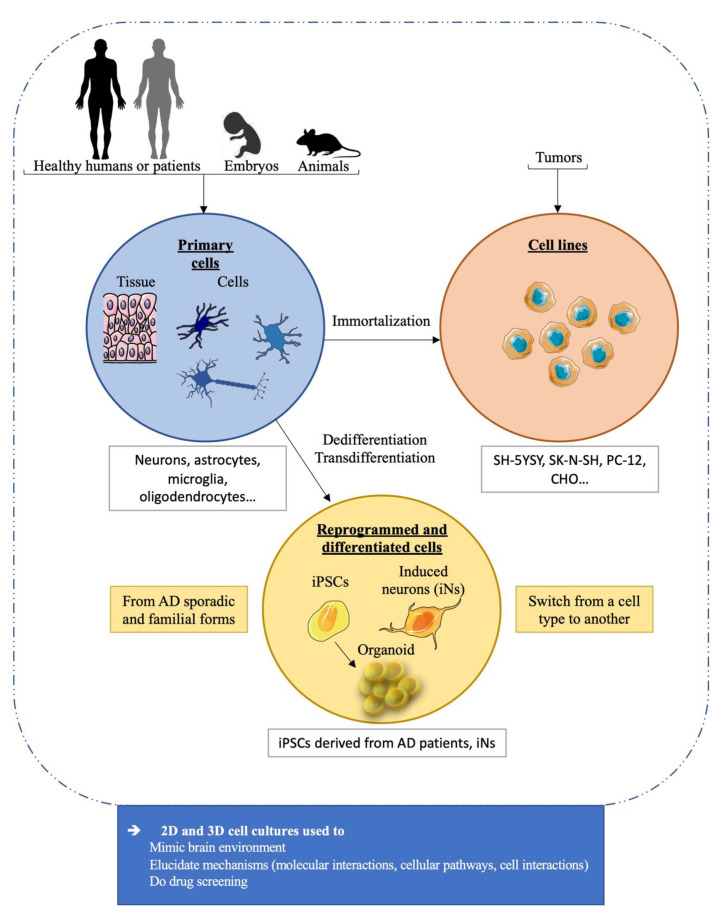
In cellulo models used in AD studies. The cell schemes are adapted from the bank image of Servier medical art smart.servier.com and are licensed under CC BY 3.0. Abbreviations. iPSCs: induced pluripotent stem cells; iNs: induced neurons.

Cell-free in vitro and in silico models are useful but often limited to the study of a simple mechanism. To better model AD, cells represent an alternative, since, as a whole living system, they allow for more complexity. They can give information on the mechanisms induced after amyloid and tau aggregations, such as toxicity, inflammation, and neurodegeneration, with the activation of several cellular pathways. All the different brain cell types are important in AD; thus, AD models do not focus only on neurons but also on various cell types involved in the pathology. To study interactions between two cell types, such as neurons and astrocytes, co-culture systems were developed, as well as 2D or 3D culture methods. Indeed, the most common way is to perform 2D cultures with adherent cells growing on a surface, but recently, 3D culture methods, which mix several cell types, were improved in order to reproduce a brain-like environment. Besides, using iPSCs, the development of organoids in flasks, as mini brains, is now a reality [3]. The cells used in AD models are described in this section and are classified into three categories, i.e., primary cells, cell lines, and reprogrammed and differentiated cells (Figure 3).

### 4.1. Primary Cells

Primary cells from AD patients, healthy donors, or animals are relevant to the physiological modeling of AD, as they are not immortalized by genetic modifications, like permanent cell lines are. With primary cells, pathological features can be directly observed by comparing cells from patients with those of healthy donors [91]. Cells can also be modified to express, at physiological levels, AD-related genes with specific fAD mutations using CRISPR/cas-9 technology [3]. From an ethical point of view, it is difficult to obtain primary cells from AD patients. They are harvested from postmortem tissues, which prevents the study of the early stages of the disease [92,93]. Furthermore, the quality of primary cell cultures from human postmortem tissues is strictly dependent on the quality of the initial samples.

#### 4.1.1. Tissues

Brain tissues from AD patients or healthy donors contain a mix of different cell types, such as neurons, astrocytes, oligodendrocytes, and microglial cells. These brain samples are one of the best cell models, because they have all the cell types forming a suitable environment for studying AD pathology. These brain tissues were used to understand the hallmarks and mechanisms of AD. For instance, Zhang et al. [91] used brain tissues from AD patients to understand the mechanism by which Aβ downregulates α-amino-3-hydroxy-5-méthylisoazol-4-propionate (AMPA) receptors (AMPARs), which are postsynaptic and glutamatergic neuronal receptors. They showed a decrease of the AMPAR amount and an increase of the AMPAR ubiquitination in AD brain tissues in comparison to a control brain (see Table 2). The brain cortex was also used to extract amyloid proteins, instead of using recombinant proteins produced in *Escherichia coli*. These amyloid molecules directly isolated from human AD patients’ brains are more relevant materials. To study the toxicity of oligomers and the interactions between Aβ and tau, Jin et al. [94] precisely extracted Aβ dimers from AD-affected brains and incubated them with primary rat hippocampal neurons. This incubation induced tau hyperphosphorylation and neurodegeneration. These experiments strongly suggest that native Aβ dimers from AD patients are sufficiently neurotoxic to trigger AD features, such as tau hyperphosphorylation and neurodegeneration.

#### 4.1.2. Neurons

In many AD cellular models, neurons are used because of their key role in the AD physiopathology. Due to the difficulties associated with obtaining AD brain tissues, most of the studies use primary cortical neurons derived from rat embryos [91,94] or suckling rats [96]. The primary cortical neurons are also used in microfluidic devices or 3D cultures to better model AD, mimicking the brain environment. These studies allow for the monitoring of neuronal degeneration and cell death after the addition of amyloid peptides or tau hyperphosphorylation inducers to the system [95,97]. These cells are also interesting for the study of the AD familial form. The mutated *APP* or *MAPT* genes can be introduced with genetic tools [113]. Another way to model AD would be to use human differentiating neurons from human embryos carrying the EOAD-related mutations. This would require preimplantation genetic diagnosis (PGD), which is conducted for other diseases, such as Down syndrome or Huntington’s disease. However, the use of human embryos raises ethical concerns, and PGD is used only for some specific diseases and not yet for AD [114].

#### 4.1.3. Astrocytes

Astrocytes, the most abundant cells in the brain, are essential for neuronal functions and survival, as they express growth factors [115,116]. Limbad et al. [100] studied the role of astrocytic senescence in AD-related neuronal degeneration. Senescence is a state in which the cell cycle is definitely stopped. It is associated with a proinflammatory secretory phenotype. In their experiments, Limbad et al. co-cultured human senescence-induced astrocytes with human fetal primary neurons and observed an induction of neuronal cell death. Therefore, astrocytic inflammation is linked to neuronal degeneration. Astrocytes were also used as a drug target for the prevention of inflammation and oxidative stress. For example, an AD model was developed using primary astrocytes from rat embryos exposed to Aβ_1–42_ peptides. In this model, the aspirin mechanism was studied, showing that, at low doses, aspirin increased cell viability and decreased inflammation and oxidative stress [99].

#### 4.1.4. Microglia

Microglial cells are the brain-resident immune cells. They are the main inducers of neuroinflammation and, accordingly, of neurodegeneration in AD. Therefore, they are well studied, since their activation correlates with cognitive loss (as reviewed by Stansley et al. [102]). They were also studied to understand their role in other AD mechanisms, such as tau seeding, propagation, and clearance. While microglial cells do not produce tau, during AD, these cells are able to take up tau seeds, probably to clear the protein aggregates. Nevertheless, this clearance seems incomplete or insufficient, as the cells release some tau seeds into the extracellular medium, thus indirectly playing a role in tau propagation and dissemination [101,117].

#### 4.1.5. Oligodendrocytes

The role of oligodendrocytes in AD was also explored. These cells form the myelin sheet around neurons, playing a role in electrochemical neurotransmission. In AD models, oligodendrocytes are notably isolated from human AD patients or from nongenetically modified rat and exposed to Aβ peptides. Horiuchi et al. [103] showed that Aβ prevents myelin sheet formation in vitro, inducing oligodendrocyte damage and cell death. The Aβ cytotoxicity was decreased when oligodendrocytes were co-cultured with astrocytes, showing that the two cell types interact, leading to a protective mechanism [104]. Therefore, primary cell culture models are useful for deciphering the role of each cell type in the AD pathology, but they are a simplified version of a complex disease, which affects the interaction of different cell types. In this context, co-culture models are better than those based on a single-cell culture.

#### 4.1.6. Endothelial Cells and Pericytes—The Blood–Brain Barrier Model

Other brain cell types are endothelial cells and pericytes, which form, with astrocytes, the BBB [22]. This barrier plays a key role in AD, especially regulating the Aβ transport inside and outside the brain with the RAGE/LRP1 receptors [21]. BBB is also affected during the disease development because of an accumulation of Aβ peptides in the brain blood capillaries [118]. In the AD pathology, the entry of solute or immune cells into the brain tissues increases due to the BBB permeability, leading to neuroinflammation [110]. Therefore, it is decisive to generate a model of BBB to understand its functions and to screen efficient drugs that can penetrate into the brain [5]. One in vitro model of the blood–brain interface is based on the brain microvessel/microvascular endothelial cells (BMECs). These cells are extracted from human or animal brains [105,106,107,108,109] (as reviewed by He et al. [119]). For example, Aβ_25–35_-exposed rat BMECs were used to measure the antiapoptotic effect of a potential curative decoction. It was shown that this Buyang Huanwu decoction, belonging to traditional Chinese medicine, inhibited the Aβ_25–35_-induced endothelial inflammation and RAGE/LRP1 dysregulation [105]. Pericytes, whose functions are altered during AD, are also used to model BBB. They are derived from AD patients’ brains or from human fetuses and are exposed to Aβ peptides [111]. As BBB is composed of different interacting cells, co-culture systems are better for fully modeling its complex architecture. For instance, endothelial cells were cultured with astrocytes to study immune cell recruitment through the BBB in a context of Aβ exposure to inflammatory molecules [110,112]. As an alternative to primary culture cells, iPSCs differentiated into brain-like endothelial cells and pericytes are used to mimic the BBB in vitro (cf. paragraph 4.3., [120]).

### 4.2. Cell Lines

The main limitation of using primary cells is their inability to be cultured for a long time, unlike cell lines, which are immortalized cells from tumors or with oncogenic modifications. However, because of their immortalization, these cell lines are also less physiological than primary cells. Cell lines were thoroughly used, because they are easy to handle and to use for generation of AD models by introducing AD-related mutations through viral vectors, CRISPR/cas-9, and other editing tools (see Table 3).

#### 4.2.1. Cell Lines Derived from Tumors

SH-SY5Y cells are the most used cell line for studying AD. Isolated from a human neuroblastoma, they are cultured in 2D and 3D systems [3,5]. AD hallmarks are induced in different manners, either with the overexpression of the *APP* gene [113,122] or with the exposure to Aβ molecules or okadaic acid, an inducer of tau hyperphosphorylation [16,121,123,124,125]. These cellular models provide an experimental system in which AD mechanisms are studied and potent drugs tested [16,122,123]. A 3D culture of SH-SY5Y cells was used to model an AD-like tauopathy by overexpressing a mutated human tau and inducing hyperphosphorylation with okadaic acid [121]. Many other cell lines derived from human neuroblastomas have been used in AD studies, such as SK-N-MC [107], SK-N-SH [126], and BE(2)-M17 [127]. Tumor cells with a non-neuronal origin have also been used: (i) the PC-12 cell line derived from a rat pheochromocytoma in the adrenal medulla [40,121,128]; (ii) the 7W CHO cells derived from a Chinese hamster ovary and stably expressing the human *APP* gene [129,130]; and (iii) the CALU-3 cell line, isolated from an adenocarcinoma of the serous epithelium of the human lung and exhibiting properties similar to those of the nasal–brain barrier [131,132].

#### 4.2.2. Immortalized Cells

Some primary cells were immortalized by transduction to facilitate their handling. Transduction consists in introducing an oncogene into the genome with a viral vector. For instance, a neural stem cell line (ReNcell VM), derived from a human fetal brain, was immortalized by transduction with the *myc* oncogene. As stem cells, the ReNcells VM have the advantage of differentiating into neurons and other glial cells, which is very suitable for modeling AD [133,134] (review: [140]). ReNcells VM were notably transduced a second time to express the *APP* gene with two AD-related mutations (Swedish and London) and used to produce neurons and astrocytes in 3D co-cultures. With this model, Choi et al. [134] succeeded in inducing the main AD hallmarks with Aβ accumulation and tau aggregation in vitro. The ReN-derived neuron and astrocyte model was even improved by adding immortalized microglia to the 3D culture [133]. Microglial cells were immortalized by the Simian virus 40 T antigen. With this triculture system, Park et al. [133] mimicked a brain environment displaying pathological AD features. 

Human and animal brain endothelial cells can also be immortalized with viral vectors to study BBB during AD (human cells: [135,136]; mouse cells: [137]). Cell types that do not originate from the brain are also used to model the pathology. HEK293, which are human embryonic kidney cells immortalized with an adenovirus, were notably used to model tau hyperphosphorylation and aggregation [138,139].

### 4.3. Reprogrammed and Differentiated Cells

With major advances in developmental biology, knowledge on cell differentiation has increased, notably elucidating the differentiation factors involved in cell lineages. It has brought tools to engineer cells, since it is now possible to dedifferentiate any cell type into a pluripotent stem cell with the Yamanaka factors [141]. The dedifferentiated cells are then called iPSCs. They are indistinguishable from embryonic stem cells and can be differentiated again in a chosen cell type when using the appropriate factors [142]. With this tool, we can develop all the brain cell types in order to study brain diseases through co-culture systems [93,142]. When the initial cells (often fibroblasts) come from fAD or sAD patients or are genetically modified to express AD-related mutations, the iPSCs-derived cells are used to study the AD hallmarks in 2D or 3D cultures (iPSC development from fAD patients [143,144], 3D culture; sAD patients [145], 3D culture; fAD patients [93,146,147] and iPCS from fAD patients, 2D culture [148]) (Table 4). Auboyer et al. [143,144] developed two iPSCs from AD patients with different mutations, one carrying the APPD694N and the other the PS1G217D. Recently, Rouleau et al. [147] developed a 3D neural tissue with human iPSCs from healthy or AD donors. This neural tissue can remain viable for up to 2 years, providing a suitable cellular model for long-term studies. Other brain cells, such as astroglial cells or BBB-composing endothelial cells, are also derived from iPSCs and are used to study AD [120,149,150,151]. Neural progenitor cells (NPCs), which play a key role in neurodevelopment, are another brain cell type derived from iPSCs [152,153]. The use of NPCs is very interesting in connection with the developmental hypothesis of the AD pathology. Indeed, Arendt et al. [154] hypothesized that exogeneous and endogenous events, such as pesticide exposure, dysregulate the NPC pool during early life stages, and these impairments could lead to the AD pathology later in life [154,155,156]. Therefore, NPC studies on potential dysregulating factors would help in understanding the role of neurogenesis in AD development. iPSCs are also used to develop organoids, which are organ-like cell clusters composed of all the cell types of an organ but with a disorganized structure [5]. Brain-like organoids with AD mutations in the *APP* or *MAPT* genes, or those exposed to amyloid molecules, are currently used to model AD ([5,157]; iPSC-derived organoid with fAD mutations [158,159]). However, one limitation of iPSCs is that reprogramming clears the cell age, leading to cells with fetal properties, which is not ideal for studying this ageing-related disease. One solution could be the induction of ageing properties with progerin overexpression or telomer shortening induced by telomerase inhibition [93]. Another method is transdifferentiation, which is the reprogramming of one somatic cell type (astrocytes or fibroblasts) to another (neurons) while retaining the epigenetic marks [160]. These cells are called induced neurons (iNs) [93,161]. The reproducibility is another key point when using iPSCs or iNs, since potential non-neuronal characteristics can persist after reprogramming. For example, the iPSCs from sAD patients have detectable differences from one patient to another and even between the different iPSC clones from the unique origin cell. Therefore, it is important to have a large set of sAD cell lines to fully understand the sAD pathology [93].

## 5. In Vivo Models of AD

An ideal in vivo model of AD should have all the physiopathological features and behavioral characteristics of AD patients, as well as reproducibility and robustness [163]. However, none of the current developed in vivo models is able to entirely reproduce the AD pathology. There are two forms of AD: the familial form due to mutations affecting 1% of patients and the sporadic form due to several risk factors affecting 99% of patients. Most of the models are based on genetic mutations observed in familial AD, since sporadic models are difficult to generate. Three types of models are distinguishable: genetic, interventional, and natural models [163]. In vivo models were first used to understand AD mechanisms, but above all, they were used to test drugs preventing cognitive deficits for therapeutic interventions. Currently, with the failure of clinical trials and no efficient treatments, in vivo models are still used to identify new innovative drugs, although rodent models are now being reconsidered. An alternative therapeutic strategy is to develop prevention, focusing on AD risk factors, such as diabetes, hypertension, neuroinflammation, and nutrition. Thus, these new therapeutic strategies aim at preventing the aggravating effects of risk factors for the AD pathology before the development of cognitive deficits [164]. In this section, the most commonly used and representative in vivo AD models are inventoried, from the simple to the more complex ones.

### 5.1. Caenorhabditis elegans, Drosophila melanogaster, and Danio rerio

#### 5.1.1. *Caenorhabditis elegans*

*Caenorhabditis elegans* is a small, free-living nematode, which is used as an invertebrate model. This worm presents a lot of advantages as a model (Table 5). *Caenorhabditis elegans* is small and has a high offspring number and a short lifespan. Its genome is entirely sequenced and encodes 65% of the human disease-associated genes. In addition, genetic tools are available to generate transgenic individuals. Thus, even if *C. elegans* is an invertebrate, this organism is well recognized in studies of ageing, neurodevelopment, neurodegenerative diseases, and other human diseases [165] (Table 5). *C. elegans* does not naturally have amyloid and tau aggregates, and this is due to different specificities. The APP protein homologue, APL-1, in *C. elegans* does not contain the Aβ sequence, and *C. elegans* does not have the β-secretase, the enzyme responsible for the cleavage of the APP protein into aggregative Aβ peptides. Therefore, most models of *C. elegans* are transgenic or either express human Aβ peptides to reproduce amyloid aggregation or human tau to induce hyperphosphorylation. One model of *C. elegans* was developed to express both the human wild type tau protein and a specific hyperphosphorylated mutant tau in neurons. The latter had mutations in a proline-rich region, leading to AD-related functional deficiencies [166,167]. As a consequence of expression of human tau proteins, this *C. elegans* model exhibited an age-dependent uncoordinated locomotion. However, according to Shen et al. [165], the mutant tau proteins used in transgenic animals are more associated with the tau pathology, frontotemporal dementia, and Parkinson’s than with the AD pathology. The authors hypothesized that AD models might not be based only on mutated tau proteins. To produce amyloid aggregates in *C. elegans*, human Aβ peptides are either constitutively expressed under specific promoters or can be induced by a temperature of 23 °C, thanks to a specific *C. elegans* system named the mRNA surveillance system [168,169]. The most commonly used promoters are the pan-neuronal and muscle promoters. For example, Sultana et al. [170] engineered transgenic *C. elegans* to express human Aβ_1–42_ peptides under the body wall muscle promoter. As they wanted to focus on the molecular processes occurring during the development of the pathology, they measured the level of protein oxidation due to the toxicity of oligomers and amyloid deposits. They compared their results with other AD models, showing the relevance of *C. elegans* as a complementary model that is able to give information on molecular interactions and cellular pathways related to AD. In an Aβ-expressing *C. elegans* model, transforming growth factor β (TGF-β) and associated transcription factors were studied to elucidate their role in the pathology. In this AD model, the loss of Sma-9, a transcriptional cofactor of the TGF-β pathway, increases Aβ deposits. As Sma-9 also plays a role in ROS production and neuroprotection, it is a potential drug target for the prevention of AD neurodegeneration [171]. More generally, TGF-β, proteostasis, and stress-related pathways are affected during AD, and their modulation by drugs curbs the AD symptoms and pathology progression [165]. Thus, in addition to the study of AD-related molecular and cellular mechanisms, the *C. elegans* model is largely used for drug screening.

#### 5.1.2. *Drosophila melanogaster*

The fly, *Drosophila melanogaster*, can be used as a human disease model, because its genome shares 70% of the human disease-related genes (Table 5). *Drosophila* expresses the orthologue genes of APP (APP-like gene, *Appl*), α-secretase, β-secretases (a fly gene family), γ-secretase, and neprilysin [172,181]. When the fly Appl and β-secretase proteins are overexpressed, *Drosophila* produces neurotoxic Aβ peptides. However, there is no significant similarity between the human and the *Drosophila* genes [181]. For this reason, humanized *Drosophila* models were also developed. One humanized *Drosophila* model notably expressed two human proteins, β-secretase and APP. This expression led to the production of Aβ_1–40_ and Aβ_1–42_ peptides and amyloid aggregates and to the reduction of the fly motor reflex behavior, memory, and lifespan [172]. While *Drosophila* AD models are not able to produce tau aggregates, tau and Aβ interactions were also studied in these models. For example, flies expressing the human-mutated APP in a tau knock-out background showed a less toxic effect of the Aβ peptide in comparison to flies expressing endogenous tau. This means that the Aβ toxicity is also due to tau [173]. The Gal4/UAS system, a genetic tool well known in *Drosophila* (explained in Tsuda et al. [174]), was also used to specifically express Aβ or tau in certain tissues. With the OK6 promoter, tau was specifically overexpressed in motoneurons to study its role in axonal functions [182]. With the Elav promoter, Aβ42 was overexpressed in all the neurons, decreasing the crawling ability and lifespan of flies [183]. Thus, *Drosophila* models show some of the AD hallmarks, i.e., Aβ aggregation, tau hyperphosphorylation, impaired synaptic activity [175], and neurodegeneration [176], but they cannot clearly recapitulate the disease, as seen in the previously described models. The *Drosophila* model is more useful as a tool for screening a library of drugs to find a cure for AD than for understanding AD mechanisms. Indeed, a high drug screening can be realized, with 100 to 500 screened molecules per month [174]. Liu and colleagues [177] identified five antioxidative plant extracts that suppress the Aβ-induced lifespan reduction through a decrease in ROS production. Another drug-screening study was described for the prevention of memory deficits. Among the interesting potential molecules, some were already identified and tested in mouse models [184]. Therefore, *Drosophila* models can be used in interdisciplinary studies to cross-validate potential therapeutics before clinical trials.

#### 5.1.3. Danio rerio

The zebrafish, *Danio rerio*, is a vertebrate used in laboratories as a powerful disease model [178,185]. Indeed, this fish is the only vertebrate that has transparent embryos and larvae, allowing researchers to conduct a lot of real-time fluorescent imaging, especially with the advent of the microscopy techniques in recent decades [186]. Therefore, the development of the nervous system of the fish is simple and well-characterized [178]. In addition, a zebrafish couple generates a lot of progenies. Zebrafish are easy to breed, the genome is fully characterized, and behavioral studies can be monitored through many assays using the zebra box [187,188,189,190]. Zebrafish are also used in high-throughput drug screening, as the tested drugs can be diluted in water. With all these advantages, zebrafish models were developed to mimic the AD pathology (Table 6). A lot of AD-related genes were also identified in this organism, such as two *app* orthologues, encoding the APPa and APPb proteins. These proteins present a high homology with the human APP protein. For example, APPa shares an 80% homology with the part of the APP protein corresponding to the Aβ region [191]. Zebrafish also have the *psen1*, *psen2*, *nicastrin*, *pen2*, and *aph1* genes, which encode for proteins composing the γ-secretase complex [178]. The PSEN1 protein is very important in the AD pathology, as 180 mutations of *PSEN1* are associated with fAD. PSEN1 also interacts with several cellular pathways involved in tau phosphorylation and calcium homeostasis. In the amyloid hypothesis, the interaction between PSEN1 and tau also explains the link between the increase and aggregation of Aβ and tau hyperphosphorylation [192]. Transgenic or knockdown zebrafish can also be engineered with genetic tools, such as targeting induced local lesions in genome (TILLING), knocking down one gene expression, or morpholino antisense oligonucleotides, which blocks transduction or splicing [178]. For example, the zebrafish genome was modified to express a mutant tau, usually associated with frontotemporal dementia [179,193,194]. In this tauopathy model, inhibitors of GSK3β, which is one of the kinases responsible for tau phosphorylation, were screened [178,179,180]. Thus, even if knowledge of AD genes in this model is lacking, zebrafish is a promising tool for understanding AD mechanisms and conducting drug screening.

### 5.2. Mouse and Rat Models

Many studies were conducted with mice and rats (Table 5). The preferential use of these rodents, compared to other in vivo models, and the accumulated knowledge explain why mice and rats are still very common in the research field of AD. As of 2021, 189 mouse and 16 rat models exist [208]. All the models are not detailed here, but relevant examples are presented in this section.

#### 5.2.1. Transgenic Mouse and Rat Models

The most frequent mouse models are transgenic animals. While the mouse *App* gene is 97% homologous to the human gene, their differences, especially in the Aβ peptide encoding part, prevent amyloid aggregation in wild-type mice. To deal with this limitation, researchers inserted the human *APP* gene inside the mouse genome [92]. For instance, the commonly used J20 transgenic mouse model overexpresses the human *APP* gene in neurons, with two AD mutations, i.e., the Swedish and the Indiana mutations, which increase the cleavages by the β- and γ-secretases, respectively [195]. These mice develop amyloid plaques and have an impaired neurotransmission, neuronal loss, and neuroinflammation, as well as cognitive impairment [196]. However, they do not develop NFTs formed by hyperphosphorylated tau. Therefore, researchers generated more aggressive models in order to induce both tau and Aβ aggregation hallmarks. For instance, the 5xFAD mice express two human transgenes, the *APP* gene, with three fAD mutations, and the *PSEN1* gene, which encodes for a subunit of the γ-secretase, with two fAD mutations [198]. However, even with all these familial AD mutations, these mice do not develop NFTs and have similar symptoms to J20 mice but develop the pathology much faster. For instance, Aβ aggregates are detectable at 2 months instead of 4-6 months for J20 mice. It is only when the *MAPT* gene was expressed with a tauopathy-associated mutation, increasing the quantity of the four-repeat tau isoform, that the first observations of tau hyperphosphorylation and pretangle formation were reported in mice [209]. Thus, with the expression of the mutated human *MAPT*, *APP*, and *PSEN1* genes, the LaFerla team succeeded in obtaining 3xTg mice developing both amyloid plaques and NFTs [199]. While this mouse model develops the two main hallmarks of AD, cognitive impairments appear before protein aggregation, unlike in humans [210]. Recently, these discrepancies between the human disease and many transgenic AD models have led to the idea that gene overexpression under strong promoters is not the best way to induce an AD-like phenotype in mouse models. Then, the challenge was to generate more physiological models [92]. For example, APP^NL-F^ knock-in mice still express the mutated human *APP* gene but at the physiological level, as the inserted gene is under the mouse endogenous *App* promoter. Thus, the gene is expressed in the suitable cell-type with the appropriate timing [200]. Admittedly, these mice do not develop NFTs but amyloid plaques, and then, in the later stages, they exhibit cognitive impairments. Even if all the features of AD are not present in this model, the evolution of the mouse pathology is closer to the human one.

Similar to mice, rats do not naturally develop AD, and the main rat models are, therefore, transgenic. Rats present the advantage of being physiologically closer to humans than mice are. Their behavioral phenotype is more sophisticated than that of mice and well-characterized [92]. Moreover, contrary to mouse models, a transgenic rat model develops NFTs, in addition to amyloid plaques, with the expression of only the endogenous rat tau protein and without the need to express the mutated human *MAPT* gene. These transgenic rats, named TgF344-AD, express the mutant human *APP* and *PSEN1* genes and exhibit neuronal loss, gliosis, and cognitive impairments [205]. The limitations of transgenic rat models are the same as those for transgenic mice with the promoter dependency. Therefore, more physiological knock-in rat models were also generated, for example, models expressing AD-associated risk variants involved in sAD. The Triggering Receptor Expressed on Myeloid Cells 2 (TREM2) knock-in rats are notably used to study the role of microglia during the disease development [206]. However, as rats are less easy to handle than mice, complete characterizations of these transgenic rat models are required before their use can be validated as a relevant model for the AD pathology.

#### 5.2.2. Interventional Mouse and Rat Models

Since genetic models are often aggressive and do not recapitulate all the disease hallmarks but only a few of them, other models, named interventional models, were developed [211]. Regarding the “injection” models, instead of inducing an increase of the quantity of Aβ peptides with the insertion of human transgenes, Aβ peptides, synthetic or isolated from patients, were directly injected into the brain of mice. This injection induces neurodegeneration and neurotransmission impairments, with cholinergic deficits, as well as an activation of inflammation actors. In Aβ-injected mice, adult neurogenesis in the subventricular zone (SVZ) is disturbed, as shown by Sotthibundhu et al. [212]. They extracted and cultured SVZ neurons from Aβ_1–42_-injected mice and observed a neurogenic effect, with an increased proliferation and differentiation of progenitor cells [201,212]. Other molecules were also injected, such as receptor antagonists to specifically induce a cholinergic deficit [211] or inducers of insulin resistance, such as streptozotocin [202]. Indeed, insulin resistance leads to the development of a lot of AD hallmarks. Streptozotocin-injected mice show cholinergic deficits, oxidative stress, neuroinflammation, and neurodegeneration. Thus, the streptozotocin–induced model represents an interesting model of the sporadic AD pathology, related to human suffering from chronic diabetes and presenting a higher risk of developing AD during ageing. Olfactory bulbectomy (OBE) is another interventional model of AD. Removing the olfactory bulb (OB) induces an AD-like physiopathology, with neurodegeneration in specific AD-affected regions, as well as neuroinflammation and an increase in Aβ levels associated with memory impairments. As OB is connected to other brain regions through nerves, OBE induces a deafferentation process, leading to all these AD-like consequences [203,211,213].

#### 5.2.3. Natural Mouse and Rat Models

Natural models are also used to study AD. For instance, senescence-accelerated mouse P8 (SAMP8) animals develop a spontaneous spongiform neurodegeneration with an excess in APP production and oxidative damage. Their memory is also impaired, and the amyloid peptide outflow from the brain is decreased [204]. An inflammatory amyloid vicious circle is then set up. Indeed, at the starting point, oxidative stress induces both mitochondrial dysfunction and Aβ accumulation, but the mitochondrial dysfunction also leads to an Aβ accumulation, which, in turn, increases the mitochondrial dysfunction through a positive-feedback loop [211].

A second natural AD model based on accelerated senescence is the OXYS rat model. These rats develop most of the AD hallmarks without any familial AD mutations. The explanation is the mitochondrial cascade hypothesis, which claims that a mitochondrial dysfunction is responsible for sAD rather than Aβ overproduction. A decrease in ATP synthesis and the presence of oxidative stress induce Aβ peptide overproduction and neurodegeneration [214]. In OXYS rats, the number of mitochondria and the activity of respiratory chain complexes decrease in correlation with the development of AD signs. Moreover, mitochondrial DNA deletions, a marker of oxidative stress, appear before Aβ overproduction. More generally, in OXYS rats, the expression of genes involved in neuronal plasticity, the immune system, apoptosis, protein phosphorylation, oxidative stress, hypoxia, and calcium homeostasis is changed before the development of AD features [207].

### 5.3. Other Mammals as Interventional or Natural Models

Other mammals are used to study AD as interventional or natural models, such as rabbits; octodon degus, an animal close to guinea pigs; and dogs (see Table 7). The injection of aluminum maltolate in aged rabbits notably induces an AD-like neuropathology, with amyloid deposits, NFT formation, and neurodegeneration [215,216]. In addition, specific food, such as a cholesterol-enriched diet and copper-containing drinking water, also induces AD-like neuropathological changes, such as neurofibrillary degeneration in rabbits. These interventional AD models are used to test drugs improving AD-like symptoms [217]. However, rabbit models of the AD pathology are quite different from the human pathology. For instance, in rabbits, NFTs are only formed with single straight filaments, composed of unphosphorylated tau proteins, instead of paired helical filaments, composed of hyperphosphorylated tau proteins in humans [215,216]. Besides, memory assays are not available in rabbits to unsure that the animals develop a full AD pathology.

Octodon degus and dogs, which develop an AD-like physiopathology with age, belong to AD natural models. An affected octodon degus shares a lot of AD hallmarks, such as an extracellular accumulation of amyloid peptides forming plaques, intracellular accumulation of tau, astrocytosis, synaptic changes, and memory impairments [218,219]. However, this model presents some limitations, because not all the animals in the colony develop the disease, and among those developing the pathology, some variabilities were shown, rendering this model quite inconsistent. Regarding aged dogs, they develop diffuse plaques, contrary to the compact human amyloid plaques. No neurofibrillary tangles are present, but only pretangles in certain aged and demented dogs. The dogs also develop a cortical atrophy and cognitive impairments. The limitations of the AD model in dogs are related to their long lifespan and the lack of consistency between the pathologies of the different animals contracting the disease [92,220].

Nonhuman primates (NHPs) are other mammals used to naturally model AD, as they develop AD-like features with age. NHPs are the genetically and anatomically closest animals to humans, with, for example, a 100% homology in the Aβ sequence [3,92]. They have a complex, well-studied, and characterized behavior and have advantages in terms of brain imaging studies due to their large brain. In addition, brain ageing in NHPs involved structural and biochemical changes similar to those associated with human brain ageing. NHPs are categorized into four groups: great apes, old world monkeys, new world monkeys, and prosimians [92,221].

The great ape group includes chimpanzees, gorillas, and orangutans. Little research has been conducted on them because of their long lifespan and ethical concerns. Aβ accumulates in the brain of aged animals, leading to plaque formation and, above all, cerebral amyloid angiopathy (CAA). The latter is a disease associated with AD and characterized by an Aβ accumulation in brain blood vessels [92,222,223,224]. The formed plaques are mostly diffuse and less abundant, compared to human plaques. The human tau sequence has a high homology with the great ape tau; however, great apes do not develop NFTs, except in very rare cases. Individuals show mild memory impairments with age; however, these deficits are equivalent to those associated with normal ageing [221]. Therefore, great apes present AD physiopathological features with age, but they do not clearly develop the disease [225].

Old world monkeys are more studied than great apes, especially the rhesus monkeys. The latter accumulate Aβ in the cortex during ageing [226], with the same spatial localization [227,228] and the same quantity or more than that accumulated in the cortex of humans [229]. This accumulation induces the formation of many diffuse amyloid plaques and the development of CAA. It also induces gliosis. Nevertheless, rhesus monkeys do not develop an AD-like pathology, as they do not show neurodegeneration, synapse loss, memory impairment, or tauopathy [230]. Zhang et al. [226] explained these discrepancies with the human pathology by a difference in the composition of Aβ oligomers. They analyzed the composition of the Aβ oligomers of aged rhesus monkeys through a comparison to human oligomers, observing that rhesus monkeys did not display Aβ dimers, which underlines their important role in the development of the human pathology. They also suggested targeting these Aβ dimers in future therapeutic strategies.

Among the new world monkeys, squirrel monkeys also accumulate amyloid peptides in their brain, leading to CAA [231,232]. They also develop amyloid plaques that are either diffuse or compact. CAA and amyloid plaques are composed of the same Aβ species present in human patients. However, squirrel monkeys do not develop NFTs. Few studies are available on the cognitive decline of new world monkeys. The available data showed a moderate age-related cognitive impairment [233,234]. Thus, squirrel monkeys, and more generally, new world monkeys, do not develop AD [221].

The most frequently studied prosimian in the research field of ageing and AD is the grey mouse lemur. They age in a similar way to humans, with the same physiological changes and age-associated cognitive declines. During ageing, 5% of the total colony show amyloid plaques in the brain [235,236,237]. Interestingly, an interindividual variability to pass cognitive tests was observed among aged lemurs with amyloid plaques, leading to subgroups among them, with weakly and highly affected individuals. The subgroup with a high cognitive decline was correlated with a high Aβ burden in the brain [238]. Only 1% of the colony developed both amyloid plaques and NFTs. Thus, very few individuals of the colony seem to be able to develop a natural AD–like pathology [221,236,239,240]. Nevertheless, the grey mouse lemur is a promising animal model compared to other NHPs. Their lifespan is relatively short (8–12 years), and they are small; mature after one year; and give birth, after a 2-month gestation, to one to three offspring by litter [241]. However, the very small percentage, i.e., around 1%, of the individuals showing both amyloidosis and tauopathy remains a major limitation for the use of grey mouse lemurs [236]. One strategy could be to identify AD-affected lemurs earlier, select AD-affected progenitors, and perform a cross-reproduction of the progeny of AD-like individuals. This process could enrich the colony of AD-affected animals. Alternatively, a solution could be the enrichment of colonies with progenies of the most affected individuals or the development of genetic tools to study transgenic animals with fAD mutations [89,242].

Thus, NHPs show Aβ aggregates and CAA with age but do not develop AD. While NHPs are phylogenetically the closest animals to humans, the disease seems specific to our species. This limitation, along with the cost, the ethical concerns, the small number of individuals available for studies, and the lack of genetic tools, results in the rare use of NHPs as AD models.

## 6. Discussion

Since different in vitro, in cellulo, and in vivo models are available for the study of AD, the current challenge is to identify and select models that provide the optimum information for a disease-specific aspect. Many publications report the use of multiple models to cross-validate results at different levels of investigation. Concerning in vitro studies, a technological barrier was crossed at the end of the 1990s with the formation of fibrils in vitro from synthetic Aβ peptides and recombinant tau proteins. Recently, with the development of cryo-electron microscopy technology, a new barrier was overcome by allowing for the direct analysis of fibrils isolated from amyloid plaques of Alzheimer’s patients with an atomic resolution. These fibrils showed structural differences, compared to recombinant fibrils, highlighting the limitation of previous structural studies. A next challenge would be to characterize many more fibrils from Alzheimer’s patients, both the familial and sporadic forms. This will allow for a better understanding of the “strains” of Alzheimer’s disease, their mechanisms, and the associated pathophysiological phenotypes. In addition, based on these recent discoveries, innovative in silico models of the entire human brain are now available for the study of the protein aggregate diffusion during AD, which will be helpful for disease prognostics when associated with imaging techniques [59,60]. However, in vitro models are not integrated systems; thus, in cellulo models are complementary and intermediate tools between in vitro and in vivo models. For decades, the cell culture models used to study Alzheimer’s disease had the advantage of easy handling, but the limiting factor was the lack of representativeness regarding the pathophysiology of the disease. Indeed, those cell lines did not develop amyloid plaques nor NFTs. A technological breakthrough was made in 2014 with the generation of iPSCs from the fibroblasts of Alzheimer’s patients. With this tool, it was possible to characterize both the familial and sporadic forms of the disease, while no model was yet available. Three-dimensional cultures of iPSCs derived from AD patients allow for the appearance of Aβ plaques and NFTs. Due to ethical issues regarding animal experimentation, it is now the perfect time for the development of the “mini brain” cutting edge tool, also called organoids, mimicking the brain environment and generated from patients’ cells. With this innovative tool, the disease will be better modeled, especially the sporadic disease. Understanding sAD is essential, since the vast majority of AD patients, i.e., 99%, are affected by this form of the disease [2]. However, iPSCs and organoids are costly and not easy to use. Future achievements will be the improvement of the protocols for handling these cells and allowing for their use in automated high-throughput screening.

Despite all the advantages of these models, one cannot completely free oneself from in vivo models. Indeed, they provide extra information about cognitive impairments and memory deficits that are impossible to study using cells, tissues, or organoids. Many animal models have been developed to mimic the disease, but none of them are able to fully recapitulate all of its characteristics. This may be related to the absence of ApoE in these models, which is a lipoprotein specific to humans and a well-known risk factor for the disease.

NHP models of AD would probably be the closest to the human disease because of the genetic proximity between humans and primates, but the disease seems specific to humans, as very few NHPs (1%) develop an AD-like pathology [236]. The major pitfalls, including the cost of the experiments, ethical concerns, time-consuming experiments, specific NHP tools that need to be developed, and the low numbers of individuals available for each experimental group, are too restrictive for basic research. A breakthrough would be the generation of transgenic AD NHPs. In particular, the grey mouse lemur would be suitable for transgenesis due to its small size, shorter lifespan, and its ability to spontaneously develop an AD-like pathology [89,242]. 

As studies with NHPs remain extremely difficult to conduct, medical research is now focusing on human patients to circumvent experimental models. Remarkably, since 2012, the Dominantly Inherited Alzheimer Network (DIAN) was created in an international effort to find a cure for AD. The purpose of the network is to collect information and tissues, following standard protocols, on individuals from families with autosomal-dominant AD in order to identify early biomarkers and treatments and accelerate the knowledge on fAD [243,244].

## 7. Conclusions

To choose the best model among the vast panel of existing AD models, it is necessary to precisely define the objectives and make some compromises. Last but not least, the majority of the models used are fAD models, and this could explain the failure to identify a treatment for sAD patients, as sporadic AD has multifactorial origins. Currently, the best way to understand the disease is to study AD patients, as the disease is specific to humans and is not found with all its characteristics in other natural or generated models. However, since access to human patient samples remains a limiting factor, breaking down barriers is required to accelerate research development and reach milestones in pharmacology. In addition, the “omics” technologies (proteomics, transcriptomics, metabolomics, etc.) will be precious tools for increasing our knowledge of Alzheimer’s disease in the future. The data acquired via the “omics” will allow us to develop early diagnoses based on the identification of early AD biomarkers, which is crucial for maximizing the efficiency of treatments [245,246,247]. Finally, even though drug development remains urgent, another field is now emerging, focusing on the prevention and identification of agents causing the disease. The idea is to tackle the roots of the problem and slow down and/or stop the development of AD, thus preventing patients from reaching the most severe stages of the disease, instead of treating the consequences of AD.

## Figures and Tables

**Table 1:** In vitro models of Aβ amyloid fibril formation and the associated biophysical techniques, based on the reviews of Malmos et al. (2017), Bruggink et al. (2012), Siddiqi et al. (2019), and Kumar et al. (2017) [34,37,38,39].

Aβ Fibrillization Models and Associated Techniques				
Associated Methods	Examples	Advantages	Pitfalls	References
Additive models				
Dye-based methods	Congo Red (CR) dye	Historical dye. Can also be used on tissues.	The short beta-sheet structures are not bound. The oligomers and protofibrils are not detected.	[32]
	Thioflavin T (ThT) assays	Best tool to study amyloid fibril formation: does not affect fibril formation, linearity, availability, robustness; easy to use.	ThT does not bind specifically to fibrils but also to DNA, cyclodextrin and SDS micelles. Need to use protein-pure samples. Cannot detect early aggregates (oligomers and protofibrils). The binding affinity depends on the fibril type. Need to use complementary techniques to confirm the results.	[33,34,40,41,42,43]
	ANS dye	Useful to characterize protein folding and aggregation intermediates.	Not specific to fibrils. Any protein with a hydrophobic region folded in the protein core has a fluorescent intensity.	[35,36,38]
Antibody-based methods	Time-resolved fluorescence (HTRF) immunoassay	Aβ peptide quantification. Sensitivity. Simple, rapid and robust method. Real-time kinetic study.	This technique requires specific antibody non cross-reacting with the different Aβ peptides.	[44,45]
	Surface plasmon resonance (SPR)	Real-time method. Study short-term or long-term aggregation kinetics (from second to hours). Study of aggregation modulators and potential drug inhibitors.	Need to know precisely which oligomer species or fibrils are bound by the antibody.	[46,47,48,49]
Microscopy and spectroscopy	Fluorescence microscopy and Fluorescence correlation spectroscopy (FCS)	Sensitivity. Real-time imaging. Small samples are sufficient. Can be used with fluorophore-coupled antibody (specificity gain). Also used to observe samples stained with Thioflavin T or ANS dyes.	Labeling can change aggregation. Autofluorescence interference.	[37,50,51,52]
Pure models				
Microscopy and spectroscopy	Time-resolved emission spectra (TRES)	Non-invasive and label-free technique. Nanosecond timescale and nanometer spatial resolution.	Difficulties for data treatment and interpretation.	[53,54]
	Turbidity, multiangle laser light scattering (MALLS), dynamic light scattering (DLS)	Label-free methods. Light scattering is very sensitive. Real-time detection.	Turbidity is not a very reliable technique. Cannot differentiate oligomer intermediates. Low resolution of light scattering techniques.	[37,38,55,56]

**Table 2:** Selection of recent key data obtained from AD primary culture models.

Primary Cells		
Cell Types	Detailed Example	References
Tissues: all the brain cell types	In AD patient brains, Aβ downregulates the neuronal receptor AMPA by increasing its ubiquitination [91].	[91,94]
Neurons	Park et al. (2015) developed an AD model based on 3D cell culture. Cultured neurons form neurospheroids in a microfluidic chip. Neurospheroids mimic a tissue with a complex neural network better than 2D-cultured neurons. Treatment with Aβ induces cell death and damages the neurospheroid network [95].	2D culture: [91,94,96] 3D culture: [95,97,98]
Astrocytes	Aβ_1-42_-exposed primary astrocytes better survive with a low dose of aspirin, probably because of a decrease in inflammation and oxidative stress [99].	[99,100]
Microglial cells	During AD, microglial cells take up tau seeds to clear the aggregates, but, because of an incomplete clearance mechanism, these cells also propagate tau seeds in other brain regions after migration [101].	[101,102]
Oligodendrocytes	Aβ prevents the myelin sheet formation in vitro, inducing oligodendrocyte damages and cell death [103].	[103,104]
BBB: endothelial cells and pericytes	The Buyang Huanwu decoction inhibits the Aβ_25–35_-induced endothelial inflammation and RAGE/LRP1 dysregulation [105].	Endothelial cells: [105,106,107,108,109] Pericytes: [110,111,112]

**Table 3:** Examples of key data obtained from AD cell line models.

Cell Lines			
Cell Lines	Associated Cell Type and Tumor	Detailed Example	References
Derived from tumor			
SH-SY5Y (also SH-SY6Y) cells	Neurons (cholinergic neurons after differentiation), derived from a neuroblastoma	In 3D culture, SH-SY5Y cells were used to model an AD-like tauopathy, induced with okadaic acid and the recombinant mutated human tau [121].	2D: [16,113,122,123,124] 3D: [121,125]
SK-N-MC cells	Neurons, derived from a neuroepithelioma	Aβ-treated SK-N-MC cells were used to find efficient drugs able to cross the BBB and rescue the degenerating neurons from apoptosis.	[107]
SK-N-SH cells	Neurons, derived from a neuroblastoma	Treatment of SK-N-SH with Aβ_25-35_ peptides was used to model AD in vitro. With this model, Gu et al. (2020) investigated genes and proteins involved in cell death during AD, identifying pathways to improve cell viability.	[126]
BE(2)-M17 cells	Neurons, derived from a neuroblastoma	Su et al. (2010) studied the role of chronic oxidative stress on tau hyperphosphorylation with a M17-based cellular stress model. They showed that stress increases tau phosphorylation in vitro and suggested a role in neurofibrillary pathology in vivo.	[127]
PC-12 cells	Chromaffin cells (modified neurons), derived from a pheochromocytoma	The neuroprotective effects of two marine-derived carotenoids was assessed with Aβ_1-42_-treated PC-12 cells [40].	[40,121,128]
7W-CHO cells	Chinese ovary cells overexpressing the human *APP* gene	7W-CHO cells were used to screen drugs able to increase the ratio between sAPPα, a neurite extending fragment, and Aβ peptides, which are neurite retractive [129].	[129,130]
CALU-3 cells	Epithelial cells, from an adenocarcinoma	CALU-3 cells were used to measure drug delivery through epithelium of a β-sheet breaker [131].	[131,132]
Immortalized with a viral vector			
ReN cells and immortalized microglial cells	Neural stem cells and microglial cells	Park et al. (2018) engineered a 3D triculture system as a model of AD neurodegeneration and neuroinflammation. They notably cultured fAD-mutated ReN cells, which are neural progenitor cells, and induced their differentiation into Aβ-overexpressing neurons and astrocytes. They also added immortalized microglial cells, completing the triculture system [133].	[133,134]
Immortalized brain endothelial cells	Endothelial cells	Endothelial cells were used to model Aβ clearance through BBB [135].	Human cells: [135,136] Mouse cells: [137]
HEK293 cells	Embryonic kidney cells	Waxman and Giasson (2011) developed a cellular model to study the induction of tau aggregation with preformed α-synuclein fibrils, another protein involved in Parkinson’s disease [138].	[138,139]

**Table 4:** Recent advances in AD reprogrammed cells.

Reprogrammed Cells		
Derived Cell Type or Tissue	Detailed Example	References
iPSCs		
Neurons	Rouleau et al. (2020) developed a 3D neural tissue with human iPSC from healthy or AD donors [147].	2D culture, iPCS from fAD patients: [93,148] 3D culture, sAD patients: [145] 3D culture, fAD patients: [146] iPSC development from AD patients: [143,144,147]
Others: astroglia, endothelial cells, NPCs	iPSC-derived human brain endothelial cells with the PSEN1 mutation show altered tight and adherent junctions and efflux properties compared to cells derived from healthy donors [151].	Astroglia: [149] Endothelial cell: [150,151] NPC: [152,153]
Organoids	Gonzalez et al. (2018) developed iPSC-derived cerebral organoids, which show a cortical organization. When the used iPSC comes from an AD patient, the developed cerebral organoid exhibits AD features such as Aβ deposition and accumulation of hyperphosphorylated tau [158].	Examples of iPSC-derived organoid with fAD mutations: [158,159] Organoid with an AD-like pathology: [162]. Reviews: [5,157]
iNs		
Neurons	Hu et al. (2015) derived fibroblasts from control and AD patients into functional neurons with chemicals [161].	[93,161]

**Table 5:** In vivo AD models in non-mammal species.

Non-Mammalian AD Models				
Models	Model Type	Advantages	Pitfalls	References
*Caenorhabditis elegans*	Transgenic	Small, easy to breed, lots of progenies. Characterized nervous system, short lifespan. Sequenced genome. Transgenic *C. elegans* can express human hyperphosphorylated tau mutant or Aβ peptides and develop some AD features. Used to study molecular interactions and cellular pathways.	Do not naturally have Aβ and β-secretase, and so, do not have amyloid aggregates. Do not naturally have tau aggregates, either. Need to be used in combination with other models.	[165,166,170]
*Drosophila melanogaster*	Transgenic	Small, easy to breed. Characterized nervous system. Sequenced genome. Have AD-related genes. Behavioral tests. Availability of genetic tools to do transgenic or knockdown models. Used for high-throughput drug screening. Transgenic flies develop AD hallmarks, such as overexpression of amyloid peptides, amyloid aggregate formation, tau hyperphosphorylation, synaptic impairments, neurodegeneration, and reduction of memory and lifespan.	AD genes are not well-characterized. Homology with human proteins but not sufficient to naturally develop the disease. Need to do transgenic models, but they do not clearly recapitulate the disease. Need to be used in combination with other models such as mouse models. Invertebrate model is very different from human than all other vertebrate models.	[172,173,174,175,176,177]
*Danio rerio* (zebrafish)	Transgenic	Small, easy to breed, lots of progenies. Characterized nervous system. Entirely sequenced genome. Have AD-related genes. Behavioral tests. Used for high-throughput drug screening. Available genetic tools for transgenic or knockdown models.	AD genes are not all well-characterized. Homology with human proteins but not sufficient to naturally develop the disease. Need to do transgenic models. Lack of data due to its recent development.	[178,179,180]

**Table 6:** Most commonly used mouse and rat models of the AD pathology.

Mouse and Rat AD Models					
Model Type	Model Name	Associated Mutation(s)	AD Characteristics	Discrepancies with AD	References
Mice					
Transgenic	J20	APP KM670/671NL (Swedish), APP V717F (Indiana)	Amyloid aggregation, neurodegeneration, neuroinflammation, cognitive impairments.	No NFTs, overexpression of mutated APP and associated fragments, deposition of amyloid plaques at 4–6 months.	[195,196]
	APPPS1	APP KM670/671NL (Swedish)PSEN1 L166P	Amyloid aggregation, neurodegeneration, neuroinflammation, cognitive impairments.	No NFTs, overexpression of mutated PSEN1 as well as mutated APP and associated fragments, deposition of amyloid plaques at 2–4 months.	[197]
	5xFAD	APP KM670/671NL (Swedish), APP I716V (Florida), APP V717I (London) PSEN1 M146L (A > C), PSEN1 L286V	Amyloid aggregation, neurodegeneration, neuroinflammation, cognitive impairments.	No NFTs, overexpression of mutated PSEN1 as well as mutated APP and associated fragments, very aggressive form, deposition of amyloid plaques at 2 months.	[198]
	3xTg	APP KM670/671NL (Swedish) MAPT P301L PSEN1 M146V	Amyloid aggregation and NFT formation, neurodegeneration, neuroinflammation, cognitive impairments.	Overexpression of mutated APP, tau, and PSEN1, amyloid plaques at 6 months, development of cognitive impairments before protein aggregation.	[199]
	APP^NL-F^ KI	APP KM670/671NL (Swedish), APP I716F (Iberian)	Amyloid aggregation, neurodegeneration, neuroinflammation, cognitive impairments, chronology of symptom development.	no NFTs.	[200]
Interventional	Aβ-injected	-	Neurodegeneration, neuroinflammation, cognitive impairments	No amyloid plaques, no NFTs.	[201]
	Receptor antagonist-injected	-	Neurodegeneration, neuroinflammation, cognitive impairments.	No amyloid plaques, no NFTs.	[202]
	Olfactory bulbectomy	-	Increase in Aβ level, neurodegeneration, neuroinflammation, cognitive impairments.	No amyloid plaques, no NFTs.	[203]
Natural	SAMP8	-	Neurodegeneration, neuroinflammation, cognitive impairments.	No amyloid plaques, no NFTs.	[204]
Rats					
Transgenic	Tg F344-AD	APP KM670/671NL (Swedish) PSEN deltaE9	Amyloid aggregation and NFT formation, neurodegeneration, neuroinflammation, cognitive impairments.	Overexpression of mutated APP and associated fragments, deposition of amyloid plaques at 6 months.	[205]
	TREM2 KI (in Human *App* background)	TREM2 R47H	Physiological expression of a sAD risk factor, production of human Aβ, only.	No amyloid plaques, no NFTs, no neurodegeneration, no neuroinflammation, no cognitive impairments.	[206]
Natural	OXYS	-	Increase in Aβ level, neurodegeneration, neuroinflammation, cognitive impairments.	No amyloid plaques, no NFTs.	[207]

**Table 7:** In vivo AD models in some mammal species.

Non-Mammalian AD Models				
Models	Model Type	Advantages	Pitfalls	References
Rabbits	Interventional	Induction of AD-pathology after brain injection of aluminum maltolate (features: amyloid aggregation, NFT formation and neurodegeneration). Non aggressive animal.	The structure of NFTs is different from human.	[215,216]
*Octodon Degus*	Natural	Development of AD-like disease with age. Aβ accumulation and plaque formation, with age. Tau accumulation. Memory impairments.	Inconsistensy from one study to another. Lack of appropriate brain map.	[92,218,219,220]
Dogs	Natural	Development of AD-like disease with age (Aβ plaques and cognitive deficits).	Diffuse plaques contrary to compact human plaques. No NFTs but pretangles. No cholinergic deficit. Long and variable lifespan. Lack of consistency.	[92,220]
*Non human Primates (NHPs)*	Natural	Development of AD-like disease with age. Genetically and anatomically closest animal to human (example: 100% homology in Aβ sequence). Well-characterized, complex, and quantifiable behaviors. Four groups of NHPs with different specificities. Similar AD symptoms: Aβ accumulation and amyloid plaque formation in the brain.	Ethical concerns. Long lifespan. Costly, few animals. Do not perfectly reproduce the human disease (often develop diffuse amyloid plaques instead of compact plaques, some primates have NFTs and others do not, mild cognitive deficits rather similar to normal ageing than to AD-induced cognitive impairments). Inter individual variability.	[3,92]

## Abbreviations

AD, Alzheimer’s disease; fAD, familial AD; sAD, sporadic AD; EOAD, early onset of AD; LOAD, late onset of AD; Aβ, amyloid β; NFTs, neurofibrillary tangles; AChE, acetylcholinesterase; FDA, Food and Drug Administration; NMDA, *N*-methyl-d-aspartate; iPSCs, induced pluripotent stem cells; APP, amyloid precursor protein; ADAM, A Desintegrin and Metalloprotease; BACE1, β-site amyloid-cleaving enzyme; PSEN1, presenilin 1; PSEN2, presenilin 2; APH1, anterior pharynx defective 1; PEN2, presenilin enhancer 2; NGF, nerve growth factor; ROS, reactive oxygen species; NO, nitric oxide; NEP, neprilysin; IDE, insulin-degrading enzyme; BBB, blood–brain barrier; LRP-1, lipoprotein receptor-related protein-1; RAGE, receptor for advanced glycation end; MAPT, microtubule-associated protein tau; GSK3β, glycogen synthase kinase 3 beta; CR, Congo Red; ThT, thioflavin T; ANS, 1-anilino-8-naphthalene sulfonate; HTRF, homogeneous time-resolved fluorescence; FCS, fluorescence correlation microscopy; EM, electron microscopy; ssNMR, solid-state nuclear magnetic resonance; MDS molecular dynamic simulations; AMPA, α-amino-3-hydroxy-5-méthylisoazol-4-propionate; AMPARs, AMPA receptors; PGD, preimplantation genetic diagnosis; BMECs, brain microvessel/microvascular endothelial cells; NPCs, neural progenitor cells; iNs, induced neurons; TILLING, targeting induced local lesions in genome; TREM2, Triggering Receptor Expressed on Myeloid Cells 2; SVZ, subventricular zone; OBE, olfactory bulbectomy; OB, olfactory bulb; SAMP8, senescence-accelerated mouse P8; NHPs, nonhuman primates; CAA, cerebral amyloid angiopathy; TGF-β, transforming growth factor β; DIAN, Dominantly Inherited Alzheimer Network.
